# Microbiological colonization of the pancreatic tumor affects postoperative complications and outcome after pancreatic surgery

**DOI:** 10.3389/fcimb.2025.1521952

**Published:** 2025-05-30

**Authors:** Esther Anna Biesel, Johanna Sundheimer, Mohamed Tarek Badr, Sara Posadas-Cantera, Sophia Chikhladze, Stefan Fichtner-Feigl, Uwe Alexander Wittel

**Affiliations:** ^1^ Department of General and Visceral Surgery, Medical Center – University of Freiburg, Freiburg, Germany; ^2^ Institute of Medical Microbiology and Hygiene, Medical Center – University of Freiburg, Freiburg, Germany

**Keywords:** pancreatic tumor, microbiological colonization, postoperative complications, mortality, survival

## Abstract

**Background:**

The patient´s microbiome has become a focal point in cancer research. Even for pancreatic cancer, alterations in the microbiome appear to influence cancer formation and progression. The aim of our single-center analysis was the examination of microbiological colonization of pancreas tissue at the time of surgery and its potential influence on complications and outcome.

**Methods:**

We prospectively evaluated patients undergoing pancreatic surgery over a three-year period from June 2018 to June 2021. We focused on the microbiological colonization of pancreatic tissue which was acquired during pancreatic surgery. Tissue samples were cultivated at our institute of microbiology. Patients´ characteristics, complications and postoperative outcome were analyzed using a prospectively maintained SPSS database.

**Results:**

Between June 2018 and June 2021, we collected pancreatic tissue samples of a total of 178 patients undergoing pancreas resections, mostly due to ductal adenocarcinoma (PDAC; 50.6%). We could cultivate bacterial or fungal species in pancreatic tissue samples of 50 of our patients (28.1%). The majority of cases were characterized by the presence of a single microbial species, but 20 patients (11.2%) showed colonization with up to four different species. Among the bacterial species detected were *Enterococcus faecium*, *Enterococcus faecalis, Escherichia coli, Staphylococcus aureus, Enterobacter cloacae* and *Klebsiella pneumonia*. We found significantly more microbiological culture growth in patients with a preoperative biliary stent (74.0% vs. 15.6%, p < 0.001). Concerning postoperative complications, we found no difference concerning pancreatic fistula, but colonization with *E. coli* was associated with a significantly higher rate of postpancreatectomy hemorrhage (30.0% vs. 8.9%, p = 0.032). Interestingly, survival of PDAC patients seems to be negatively affected by positive microbiological findings at the time of surgery, but without reaching statistical significance (p = 0.770).

**Conclusion:**

In this first analysis of our patient cohort, we could show a microbiological colonization of pancreatic tumor tissue in almost a third of our patients. There seems to be only a minor impact on postoperative complications, but long-term outcome seems to be worse in patients with a positive pancreas microbiome. Further observation is needed to evaluate the influence of the tumor microbiome on the long-term oncological outcome in PDAC patients.

## Introduction

The human body is the habitat of diverse microorganisms which – taken together as microbiome – have important metabolic functions (Frost et al., 2022). However, shifts in the composition of the body´s microbiome may lead to disease development and progression (Frost et al., 2022). During recent years, the patient´s microbiome turned in the focus of cancer research (Picardo et al., 2019), as microbiota reside on or within about 20% of malignancies (de Martel et al., 2012; Wei et al., 2019). Even for pancreatic adenocarcinoma – still one of the deadliest malignancies – alterations in the microbiome seem to influence cancer formation and progression. It could be shown that the pancreatic cancer tissue comprises a more abundant microbiome compared to normal pancreatic tissue both in humans as well as in mice (Pushalkar et al., 2018) and that selected bacteria are differentially increased in pancreatic cancer tissue, compared to the gut microbiome (Pushalkar et al., 2018). In mice, ablation of the microbiome seems to protect against pre-invasive and invasive pancreatic ductal adenocarcinoma (PDAC) (Pushalkar et al., 2018). Clinically, intratumoral microbes may influence carcinogenesis and treatment response via different mechanisms (McAllister et al., 2019). A study could show that Fusobacterium species were present in some patients with pancreatic cancer and that their presence within the pancreatic tumor was associated with a worse prognosis of these patients (Mitsuhashi et al., 2015). Geller et al. showed that specific bacteria from the Gammaproteobacteria class, including the Enterobacteriaceae and Pseudomonadaceae families, present in pancreatic tumor tissue, play a role in conferring resistance to gemcitabine, a commonly used chemotherapy drug for pancreatic cancer (Geller et al., 2017). Moreover, the tumor microbiome composition in PDAC-patients may play a role in promoting long-term survival by influencing the host´s immune response (Riquelme et al., 2019) and the presence of intratumoral microbes in long-term survivors was associated with enhanced immune infiltrates (Balachandran et al., 2017). In addition to bacterial colonization, the presence of fungi in pancreatic tumor tissue also affects the course of pancreatic cancer (Aykut et al., 2019). A high abundance and distinct composition of fungal infection was detected in both murine and human pancreatic tumor tissue when compared to normal pancreatic tissue. Moreover, antifungal therapy with oral amphotericin B led to delayed tumorigenesis and tumor growth in the mouse model and potentiated the effect of gemcitabine (Aykut et al., 2019).

The detection of microbes by polymerase chain reaction (PCR), which is performed by the trials mentioned above, is very sensitive; however, it is not able to differentiate between vital and non-vital bacteria. Even in studies evaluating the influence of different risk factors, e.g. smoking, on the pancreatic tumor microbiome, only sequencing methods are used to detect potential bacteria (Liang et al., 2024). So far, PCR and sequencing methods are the predominant methods in order to evaluate the intratumoral microbiota of different tumor entities (Xue et al., 2023), but analyses using conventional microbiological cultures of the tumor tissue are still lacking. Therefore, the aim of our single center study was a first evaluation of the vital microbiome of pancreatic tumors by analyzing bacterial and fungal colonization of patients´ tumor specimens using conventional microbiological cultures. Moreover, our aim was to analyze the potential impact of the pancreatic tumor microbiome on postoperative complications and long-term outcome of patients. In addition, it will be examined whether different microbial species correlate with specific complications after pancreas resections.

## Methods

### Patient collective and data collection

We prospectively evaluated our patients undergoing pancreatic surgery at the University Hospital Freiburg over a three-year period from June 2018 to June 2021 concerning microbiological colonization of pancreatic tissue. Pancreatic tumor tissue was extracted intraoperatively via Tru-Cut biopsy needles after the resection of the pancreas, so that there was no risk of lacerating neighboring structures. Tissue samples were cultivated for bacterial and fungal species at our institute of microbiology, including testing for bacterial or fungal resistances against special antibiotics or antimycotics. Patients´ characteristics, complications and postoperative outcome were analyzed using a prospectively maintained SPSS database. Postoperative complications such as pancreatic fistula (POPF), postpancreatectomy hemorrhage (PPH) or delayed gastric emptying (DGE) were graded by current international definitions of the International Study Group of Pancreatic Surgery (ISGPS) (Bassi et al., 2005; Wente et al., 2007b; Wente et al., 2007a; Bassi et al., 2017).

### Microbiological culture-based methods and microscopy

Pancreatic tissue samples were examined microscopically using Gram staining to detect granulocytes and bacteria. Samples were also plated on various cultural media, including Columbia blood agar (Thermo ScientificTM OxoidTM, Wesel, Germany), chocolate blood agar and MacConkey agar, followed by incubation for at least 48 h under aerobic conditions (36°C, 5% CO2). For the cultivation of strict anaerobic bacteria, yeast cysteine blood agar (HCB; in-house) was used under anaerobic conditions in a jar or plastic bags with either the Genbox ANAER (bioMérieux, Marcy-l’Étoile, France) or the Anaerocult (Merck, Darmstadt, Germany) system. Brain heart infusion broth containing 0.093% (w/v) agar was inoculated and incubated for five days.

Microorganisms were identified using matrix-assisted laser desorption ionization-time-of-flight mass spectrometry (MALDI-TOF, Bruker Daltonics, Bremen, Germany). Antimicrobial susceptibility testing of the detected organisms was conducted using the VITEK^®^2 system (bioMérieux, Nürtingen, Germany) and interpreted according to EUCAST resistance breakpoints (http://www.eucast.org/).

### Microbial genomic DNA preparation and sequencing using illumina 16S rDNA sequencing

Besides conventional microbiological culture, twenty of our PDAC tumor samples were analyzed via 16S rDNA sequencing as well. These samples were processed and analyzed using the ZymoBIOMICS^®^ Targeted Sequencing Service (Zymo Research, Irvine, CA). DNA was extracted using either the ZymoBIOMICS^®^-96 MagBead DNA Kit on an automated platform or the ZymoBIOMICS^®^ DNA Miniprep Kit (Zymo Research, Irvine, CA). Bacterial 16S ribosomal RNA gene-targeted sequencing was conducted with the Quick-16S™ NGS Library Prep Kit (Zymo Research, Irvine, CA), using primers that amplified the V1-V2 region of the 16S rRNA gene. Final PCR products were quantified using qPCR fluorescence readings and pooled based on equal molarity. The pooled library was cleaned with the Select-a-Size DNA Clean & Concentrator™ (Zymo Research, Irvine, CA), and subsequently quantified with TapeStation^®^ (Agilent Technologies, Santa Clara, CA) and Qubit^®^ (Thermo Fisher Scientific, Waltham, WA). The final library was sequenced on the Illumina^®^ MiSeq™ using a v3 reagent kit (600 cycles) with a 10% PhiX spike-in.

### Sequencing analysis pipeline

Raw fastq files were analyzed as previously described (Wetzel et al., 2023; Lichtenegger et al., 2024). In short, raw fastq files’ read quality was assessed using FastQC (Babraham Bioinformatics - FastQC) and MultiQC (Ewels et al., 2016). Further quality control measures, trimming, and analysis of Illumina short-reads were done using the DADA2 analysis pipeline (Callahan et al., 2016). Reads were trimmed after 230 base pairs and filtered with 2 and 5 maximum expected errors in the forward and reverse reads respectively, apart from the default filtering parameters. Amplicon sequence variants (ASVs) were extracted from DADA2 and were assigned to taxonomy ranks using the Genome Taxonomy Database (Parks et al., 2020) release 207. Rarefaction curves were used to estimate sequencing depth.

### Bacterial diversity and taxonomy analysis

Further analysis concerning bacterial diversity was carried out using the R programming language. The phyloseq R package (McMurdie and Holmes, 2013) was used to calculate bacterial diversity. Observed, Shannon, and inverse Simpson (InvSimpson) were used as alpha diversity indices. For beta diversity and taxonomy analysis, ASVs with fewer than ten occurrences in all samples were excluded. The microbial Bray-Curtis distance between samples was calculated with the phyloseq R package and visualized using Principal Coordinates Analysis (PCoA). Statistical analyses were conducted with the stats R package (R Core Team. R, 2013). For statistical differences in taxonomy, the Kruskal-Wallis or Wilcoxon rank-sum tests were applied to non-normally distributed variables. Categorical variables were analyzed using the Fisher exact test. Cumulative Sum Scaling (CSS) was performed using the metagenomeSeq package (Paulson et al., 2013) and applied for heatmap visualization. Visualization of samples was achieved using functions from the R packages phyloseq (McMurdie and Holmes, 2013), microViz (Barnett et al., 2021), ggplot2 (Wickham, 2009) and pheatmap (Kolde). The code for this analysis is publicly available at the following link: https://github.com/S-Posadas/Pancreatic_tumor_microbial_colonization.

### Statistical analysis

Statistical analysis concerning patient data was performed using SPSS (IBM SPSS Statistics for Windows, Version 29.0. IBM Corp., Armonk, NY, USA). After performing explorative analysis and descriptive statistics, statistical significance was examined by using chi-square tests and Fisher´s exact tests for categorical variables and ANOVA for continuous variables. Survival estimates were calculated using Kaplan-Meier curves and log-rank tests. Results with a p-value < 0.05 were considered statistically significant.

### Ethics

Data collection and analysis were performed in accordance with the Declaration of Helsinki and were approved by the local ethics committee (Ethics Committee of Albert-Ludwigs-University Freiburg, Germany, EK-No. 23-1416-S1-retro).

## Results

### Baseline characteristics and intraoperative parameters

Between June 2018 and June 2021, we collected intraoperative tissue samples of a total of 178 patients undergoing pancreas resections at the University Hospital Freiburg. Most pancreas resections were performed due to pancreatic ductal adenocarcinoma (PDAC; 50.6%). In the majority of cases, patients underwent pancreatoduodenectomies (140 patients, 78.7%). In 50 of our patients (28.1%), we found microbiological colonization of the pancreas tissue at the time of surgery; the remaining 128 samples remained sterile. Dividing the patients in two groups depending on negative (neg) or positive (pos) microbiological findings, we could find no difference concerning age (66 years vs. 68 years, *P* = 0.099), sex (female 42.2% vs. 32.0%, *P* = 0.211), preoperative ASA stadium (ASA II 27.3% vs. 30.0%, *P* = 0.723; ASA III 68.0% vs. 66.0%, *P* = 0.801) or comorbidities (91.4% vs. 88.0%, *P* = 0.487) between the groups. Moreover, we could find no difference concerning alcohol (16.5% vs. 26.0%, *P* = 0.150) or nicotine consumption (39.1% vs. 30.0%, *P* = 0.200). The rate of neoadjuvant treatment was similar in both groups (9.4% vs. 14.0%, *P* = 0.369). Preoperative parameters such as preoperative leucocytes (7100/µl vs. 7000/µl, *P* = 0.384), creatinine (0.84 mg/dl vs. 0.75 mg/dl, *P* = 0.674), international normalized ratio (INR; 1.02 vs. 1.01, *P* = 0.136) and serum amylase (28.0 U/l vs. 22.0 U/l, *P* = 0.486) did not differ between the groups, but we found a significantly lower hemoglobin (12.5 g/dl vs. 13.3 g/dl, *P* = 0.007) as well as a higher median bilirubin (0.75 mg/dl vs. 0.60 mg/dl, *P* = 0.019) in patients with a positive microbiological colonization. Furthermore, patients with a positive microbiological colonization showed a significantly longer duration of surgery (402 min vs. 359.5 min, *P* = 0.008) and needed a higher amount of intraoperative blood transfusions (140.8 ml vs. 32.8 ml, *P* = 0.028). Interestingly, the rate of advanced lymph node metastasis (N2 stadium) was significantly higher in patients with a positive microbiological colonization of the pancreatic tumor (41.7% vs. 21.5%, *P* = 0.021). For details concerning baseline characteristics and intraoperative parameters of the entire cohort see **Table 1**, for the cohort of PDAC patients see **Table 2**.

**Table 1:** Baseline characteristics and intraoperative parameters of the entire collective.

	Negative microbiological colonization of pancreatic tumor (n = 128)	Positive microbiological colonization of pancreatic tumor (n = 50)	p-value
Age, years (median, range)	66 (20 – 86)	68 (23 – 84)	0.099
Sex (n, %)			
- male	74 (57.8)	34 (68.0)	0.211
- female	54 (42.2)	16 (32.0)	
BMI, kg/m2 (median, range)	25.2 (16.2 – 43.9)	25.5 (17.3 – 64.3)	0.341
ASA stadium (n, %)			
- ASA 1	1 (0.8)	0 (0.0)	0.531
- ASA 2	35 (27.3)	15 (30.0)	0.723
- ASA 3	87 (68.0)	33 (66.0)	0.801
- ASA 4	5 (3.9)	2 (4.0)	0.977
Comorbidities (n, %)	117 (91.4)	44 (88.0)	0.487
- Coronary heart disease	11 (8.6)	7 (14.0)	0.282
- Hypertension	71 (55.5)	31 (62.0)	0.429
- Pulmonary disease	28 (21.9)	8 (16.0)	0.380
- Renal disease	14 (10.9)	4 (8.0)	0.559
- Liver disease	19 (14.8)	8 (16.0)	0.847
- Diabetes mellitus	33 (25.8)	19 (38.0)	0.107
Alcohol abuse (n, %)	21 (16.5)	13 (26.0)	0.150
Nicotin abuse (n, %)	50 (39.1)	15 (30.0)	0.200
Neoadjuvant therapy (n, %)	12 (9.4)	7 (14.0)	0.369
Bile duct stent preoperative (n, %)	20 (15.6)	37 (74.0)	**< 0.001**
Preoperative leucocytes*10³/µl (median, range)	7.1 (3.1 – 17.4)	7.0 (2.9 – 17.0)	0.384
Preoperative hemoglobin, g/dl (median, range)	13.3 (8.9 – 20.4)	12.5 (8.4 – 15.5)	**0.007**
Preoperative thrombocytes*10³/µl (median, range)	257.0 (83 – 589)	261.5 (41 – 583)	0.574
Preoperative creatinine, mg/dl (median, range)	0.84 (0.4 – 2.2)	0.75 (0.4 – 2.1)	0.674
Preoperative bilirubine, mg/dl (median, range)	0.6 (0.2 – 33.8)	0.75 (0.2 – 9.8)	**0.019**
Preoperative serum amylase, U/l (median, range)	28.0 (2.0 – 674.0)	22.0 (3.0 – 255.0)	0.486
Preoperative INR (median, range)	1.02 (0.91 – 1.85)	1.01 (0.90 – 1.20)	0.136
Indication for surgery (n, %)			
- PDAC	66 (51.6)	24 (48.0)	0.669
- periampullary carcinoma	15 (11.7)	12 (24.0)	**0.040**
- IPMN	11 (8.6)	2 (4.0)	0.290
- Chronic pancreatitis	9 (7.0)	7 (14.0)	0.144
- Neuroendocrine tumor	14 (10.9)	1 (2.0)	0.054
- Other malign	2 (1.6)	0 (0.0)	0.374
- Other benign	11 (8.6)	4 (8.0)	0.898
Duration of surgery, minutes (median, range)	359.5 (78 – 641)	402 (162 – 722)	**0.008**
Surgical technique (n, %)			
- pancreatoduodenectomy (open)	42 (32.8)	18 (36.0)	0.686
- pancreatoduodenectomy (min. invasive)	53 (41.4)	27 (54.0)	0.129
- distal pancreatectomy (open)	4 (3.1)	1 (2.0)	0.683
- distal pancreatectomy (min. invasive)	17 (13.3)	0 (0.0)	**0.007**
- total pancreatectomy	4 (3.1)	3 (6.0)	0.375
- laparoscopic enucleation	1 (0.8)	0 (0.0)	0.531
- other surgery	7 (5.5)	1 (2.0)	0.315
Transfusion of red blood cells intraoperatively, ml (mean, SD)	32.8 (217.0)	140.8 (424.7)	**0.028**
Resection margin negative (R0) (n, %)	82 (85.4)	31 (83.8)	0.813
Histopathological classification (n, %) (n = 131)			
- T1	20 (21.3)	7 (19.4)	0.818
- T2	35 (37.2)	16 (44.4)	0.451
- T3	33 (35.1)	12 (33.3)	0.849
- T4	6 (6.4)	1 (2.8)	0.415
- N0	43 (46.2)	12 (33.3)	0.184
- N1	30 (32.3)	9 (25.0)	0.421
- N2	20 (21.5)	15 (41.7)	**0.021**

p-values in bold print are statistically significant.

**Table 2:** Baseline characteristics and intraoperative parameters of PDAC patients only.

	Negative microbiological colonization of pancreatic tumor (n = 66)	Positive microbiological colonization of pancreatic tumor (n = 24)	p-value
Age, years (median, range)	66 (28 – 86)	67.5 (54 – 82)	0.302
Sex (n, %)			
- male	38 (57.6)	16 (66.7)	0.436
- female	28 (42.4)	8 (33.3)	
BMI, kg/m2 (median, range)	25.1 (17.7 – 39.1)	25.5 (17.3 – 64.3)	0.497
ASA stadium (n, %)			
- ASA 1	0 (0.0)	0 (0.0)	0.999
- ASA 2	17 (25.8)	8 (33.3)	0.478
- ASA 3	45 (68.2)	15 (62.5)	0.613
- ASA 4	4 (6.1)	1 (4.2)	0.729
Comorbidities (n, %)	59 (89.4)	21 (87.5)	0.800
- Coronary heart disease	6 (9.1)	3 (12.5)	0.634
- Hypertension	36 (54.5)	15 (62.5)	0.501
- Pulmonary disease	14 (21.2)	5 (20.8)	0.969
- Renal disease	5 (7.6)	4 (16.7)	0.204
- Liver disease	9 (13.6)	2 (8.3)	0.497
- Diabetes mellitus	19 (28.8)	7 (29.2)	0.972
Alcohol abuse (n, %)	8 (12.1)	3 (12.5)	0.961
Nicotin abuse (n, %)	26 (39.4)	7 (29.2)	0.224
Neoadjuvant therapy (n, %)	12 (18.2)	7 (29.2)	0.259
Bile duct stent preoperative (n, %)	11 (16.7)	21 (87.5)	**< 0.001**
Preoperative leucocytes*10³/µl (median, range)	7.1 (3.1 – 16.4)	7.1 (4.5 – 17.0)	0.098
Preoperative hemoglobin, g/dl (median, range)	13.2 (8.9 – 16.6)	12.1 (9.2 – 15.2)	**0.033**
Preoperative thrombocytes*10³/µl (median, range)	245 (83 – 440)	256 (142 – 583)	0.121
Preoperative creatinine, mg/dl (median, range)	0.84 (0.4 – 1.5)	0.9 (0.4 – 2.1)	0.257
Preoperative bilirubine, mg/dl (median, range)	0.85 (0.2 – 33.8)	1.1 (0.2 – 5.4)	**0.043**
Preoperative serum amylase, U/l (median, range)	22.0 (2 – 674)	21.5 (3 – 211)	0.501
Preoperative INR (median, range)	1.02 (0.92 – 1.41)	1.02 (0.94 – 1.19)	0.627
CA 19–9 preoperative, U/l (median, range)	65.3 (1.4 – 10 000)	77.3 (9.0 – 1052)	0.288
Duration of surgery, minutes (median, range)	389 (177 – 609)	423.5 (258 – 722)	0.100
Surgical technique (n, %)			
- pancreatoduodenectomy (open)	24 (36.4)	10 (41.7)	0.646
- pancreatoduodenectomy (min. invasive)	23 (34.8)	9 (37.5	0.816
- distal pancreatectomy (open)	3 (4.5)	1 (4.2)	0.939
- distal pancreatectomy (min. invasive)	5 (7.6)	0 (0.0)	0.165
- total pancreatectomy	4 (6.1)	3 (12.5)	0.313
- laparoscopic enucleation	0 (0.0)	0 (0.0)	0.999
- other surgery	7 (10.6)	1 (4.2)	0.342
Transfusion of red blood cells intraoperatively, ml (mean, SD)	54.6 (297.3)	187.5 (536.7)	0.140
Resection margin negative (R0) (n, %)	53 (82.8)	20 (83.3)	0.954
Histopathological classification (n, %) (n = 90)			
- T1	13 (20.0)	5 (20.8)	0.931
- T2	30 (46.2)	12 (50.0)	0.747
- T3	21 (32.3)	6 (25.0)	0.506
- T4	1 (1.5)	1 (4.2)	0.458
- N0	28 (43.1)	6 (25.0)	0.119
- N1	23 (35.4)	7 (29.2)	0.582
- N2	14 (21.5)	11 (45.8)	0.024

p-values in bold print are statistically significant.

### Microbiological colonization

**Figure 1:**
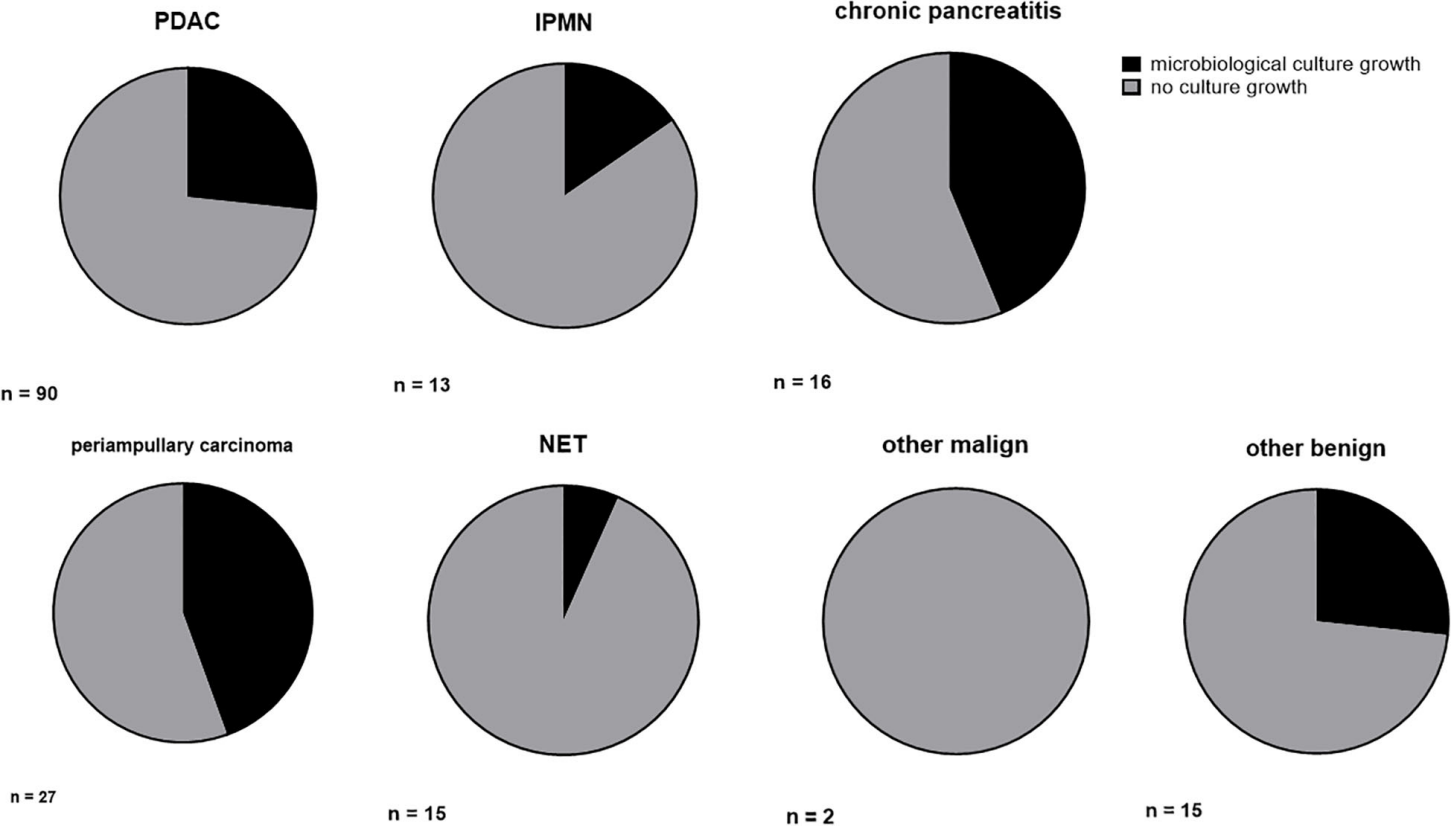
Ratio of microbiological colonization in different tumor entities. Ratio (%) of microbiological colonization in conventional culture growth. PDAC, pancreatic ductal adenocarcinoma; IPMN, intrapapillary mucinous neoplasm; NET, neuroendocrine tumor.

In 50 of our 178 patients, we detected microbiological colonization of the pancreatic tumor at the time of surgery (28.1%). Most of our patients showed only colonization with one microbiological species, but 20 patients (11.2%) revealed colonization with up to four different microbiological species in their tissue samples. Among the bacteria detected were *Enterococcus faecium, Enterococcus faecalis, Escherichia coli, Enterobacter cloacae* and *Klebsiella pneumoniae*. An overview of the microbiological findings in our patient collective is given in **Table 3**. The highest rate of bacterial colonization is found in patients with chronic pancreatitis (7 of 16 patients, 43.8%) and periampullary carcinomas (12 of 27 patients, 44.4%), even reaching statistical significance in the later (*P* = 0.040). The rate of positive microbiological colonization in the different tumor entities is shown in **Figure 1**.

**Table 3:** Microbiological species in our patient cohort.

Microbiological species	n	%
No colonization	129	72.1
***Enterococcus faecium***	4	2.2
***Enterococcus faecalis***	8	4.5
***Escherischia coli***	3	1.7
*Staphylococcus aureus*	1	0.6
*Staphylococcus warneri*	2	1.1
*Cutibacterium (Propionibacterium) acnes*	1	0.6
*Enterobacter cloacae complex*	2	1.1
*Streptococcus anginosus* (group)	1	0.6
*Klebsiella pneumoniae*	3	1.7
*Citrobacter freundii*	1	0.6
*Candida tropicalis*	1	0.6
*Actinomaces naeslundi*	1	0.6
*Bacillus* sp*ecies*	1	0.6
*Klebsiella oxytoca (Raoultella)*	1	0.6
***Escherischia coli, Enterococcus faecalis***	4	2.2
***Enterococcus faecium*** *, Hafnia alvei*	1	0.6
***Escherischia. coli*** *, Enterobacter cloacae*	1	0.6
*Enterobacter cloacae, Streptococcusanginosus*	1	0.6
*Staphylococcus epidermidis, Lactobacillus rhamnosus*	1	0.6
***Enterococcus. faecalis*** *, Enterobacter cloacae*	2	1.1
***Enterococcus faecium, Entorococcus faecalis***	1	0.6
*Citrobacter freundii*, ***Enterococcus faecalis***	1	0.6
***Enterococcusfaecium*** *, Klebsiella pneumoniae*	1	0.6
*Citrobacter koseri, Klebsiella*, ***Entorococcus faecalis***	1	0.6
***Enterococcus faecalis, Escherischia coli*** *, Klebsiella pneumoniae*	1	0.6
***Enterococcus faecium, Enterococcus faecalis*** *, Candida albicans*	1	0.6
***Enterococcus malodoratus, Enterococcus faecium, Enterococcus gallinarium*** *, Klebsiella pneumoniae*	1	0.6
*Candida albicans, Streptococcus anginosus, Enterobacter cloacae, Prevotella intermedia*	1	0.6
***Enterococcus avium*** *, Streptococcus anginosus, Citrobacter koseri, Actinomyces* sp*ecies*	1	0.6

### Postoperative complications and length of hospital stay

In our patient collective, we found a similar distribution of delayed gastric emptying (DGE), postpancreatectomy hemorrhage (PPH) and clinically relevant pancreatic fistula (CR-POPF) between patients with and without microbiological findings in the pancreatic tumor (DGE neg 39.7% vs. pos 34.7%, *P* = 0.542; PPH B/C neg 10.2% vs. pos 10.0%, *P* = 0.975; CR-POPF neg 35.9% vs. pos 34.0%, *P* = 0.808) (**Table 4**). Moreover, we found no difference between both groups concerning urinary tract infection (neg 4.7% vs. pos 4.0%, *P* = 0.842), wound infections (19.5% vs. 16.0%, *P* = 0.586), intraabdominal abscesses (12.5% vs. 12.0%, *P* = 0.927), pneumonia (7.0% vs. 4.0%, *P* = 0.450) and acute kidney failure (3.9% vs. 8.2%, *P* = 0.249) following surgery. Even concerning postoperative sepsis, we found no difference between patients with negative and positive microbiological colonization (neg 5.5% vs. pos 4.0%, *P* = 0.688), but there seemed to be a trend towards more thromboembolic complications in patients with a microbiological colonization of the pancreatic tumor (pos 8.0% vs. neg 2.3%, *P* = 0.081). There was no significant difference concerning revision surgery (neg 14.1% % vs. pos 18.4%, *P* = 0.476) or the need of postoperative interventional therapies (neg 32.8% vs. pos 36.0%, *P* = 0.686), but patients with a positive microbiological colonization showed a trend towards receiving additional conservative treatment more frequently (pos 86.0% vs. neg 73.4%, *P* = 0.074). The latter was mainly due to the preoperatively inserted bile duct stent, which leads to a routine postoperative antibiotic therapy following our hospital standards. The rate of postoperative mortality was similar between both groups (neg 2.3% vs. pos 2.0%, *P* = 0.889) as well as the length of stay of the intensive care unit (ICU) (neg median 5 days (2–38 days) vs. pos 5 days (3–41 days), *P* = 0.636) and the length of hospital stay (16 days (5–76 days) vs. 17 days (6–73 days), *P* = 0.988). Details on postoperative complications and hospital stay are summarized in **Table 4**.

**Table 4:** Postoperative complications and length of stay.

	Negative microbiological colonization of pancreatic tumor (n = 128)	Positive microbiological colonization of pancreatic tumor (n = 50)	p-value
Delayed Gastric Emptying (DGE) (n, %)	50 (39.7)	17 (34.7)	0.542
Postpancreatectomy Hemorrhage (PPH B/C) (n, %)	13 (10.2)	5 (10.0)	0.975
Pancreatic fistula (CR-POPF) (n, %)	46 (35.9)	17 (34.0)	0.808
Urinary tract infection (n, %)	6 (4.7)	2 (4.0)	0.842
Wound infection (n, %)	25 (19.5)	8 (16.0)	0.586
Thrombembolism (n, %)	3 (2.3)	4 (8.0)	0.081
Intraabdominal abscess (n, %)	16 (12.5)	6 (12.0)	0.927
Pneumonia (n, %)	9 (7.0)	2 (4.0)	0.450
Reintubation (n, %)	11 (8.6)	3 (6.0)	0.563
Sepsis (n, %)	7 (5.5)	2 (4.0)	0.688
Acute kidney failure (n, %)	5 (3.9)	4 (8.2)	0.249
Insufficiency BDA (n, %)	1 (0.8)	0 (0.0)	0.531
Revision surgery (n, %)	18 (14.1)	9 (18.4)	0.476
Postoperative interventional therapy (n, %)	42 (32.8)	18 (36.0)	0.686
Postoperative conservative therapy (n, %)	94 (73.4)	43 (86.0)	0.074
Postoperative mortality (n, %)	3 (2.3)	1 (2.0)	0.889
Length of hospital stay, days (median,range)	16 (5 – 76)	17 (6 – 73)	0.988
Length of ICU stay, days (median, range)	5 (2 – 38)	5 (3 – 41)	0.636

### Association of specific bacteria and postoperative complications

In a next step, we analyzed if a specific microbiological colonization of the pancreatic tumor was associated with postoperative complications. Here, we could find no influence of enterococcus species (neither *E. faecium* nor *E. faecalis* nor both) on postoperative complications (**Table 5**). In case of infections with *E. coli* species in the pancreatic tumor, significantly more cases of postpancreatectomy hemorrhage grade B/C (30.0% vs. 8.9%, p = 0.032) and a trend towards more clinically relevant pancreatic fistula (60.0% vs. 33.9%, p = 0.094) and wound infections (40.0% vs. 17.3%, p = 0.072) were observed (**Table 6**).

**Table 5:** Complications in association with colonization with Enterococcus species at the time of surgery.

	No Enterococcus species on pancreatic tumor (n = 151)	Colonization with Enterococcus species (n = 27)	p-value
Delayed Gastric Emptying (DGE) (n, %)	58 (38.9)	9 (34.6)	0.676
Postpancreatectomy Hemorrhage (PPH B/C) (n, %)	16 (10.6)	2 (7.4)	0.613
Pancreatic fistula (CR-POPF) (n, %)	52 (34.4)	11 (40.7)	0.528
Urinary tract infection (n, %)	6 (4.0)	2 (7.4)	0.428
Wound infection (n, %)	30 (19.9)	3 (11.1)	0.281
Thrombembolism (n, %)	6 (4.0)	1 (3.7)	0.947
Intraabdominal abscess (n, %)	17 (11.3)	5 (18.5)	0.291
Pneumonia (n, %)	10 (6.6)	1 (3.7)	0.562
Reintubation (n, %)	13 (8.6)	1 (3.7)	0.383
Sepsis (n, %)	8 (5.3)	1 (3.7)	0.728
Acute kidney failure (n, %)	7 (4.6)	2 (7.7)	0.512
Insufficiency BDA (n, %)	1 (0.7)	0 (0.0)	0.672
Revision surgery (n, %)	22 (14.7)	5 (18.5)	0.608
Postoperative interventional therapy (n, %)	51 (33.8)	9 (33.3)	0.964
Postoperative conservative therapy (n, %)	113 (74.8)	24 (88.9)	0.110
Postoperative mortality (n, %)	3 (2.0)	1 (3.7)	0.579

**Table 6:** Complications in association with colonization with E. coli at the time of surgery.

	No evidence of E. coli on pancreatic tumor (n = 168)	Colonization with E. coli (n = 10)	p-value
Delayed Gastric Emptying (DGE) (n, %)	64 (38.8)	3 (30.0)	0.579
Postpancreatectomy Hemorrhage (PPH B/C) (n, %)	15 (8.9)	3 (30.0)	**0.032**
Pancreatic fistula (CR-POPF) (n, %)	57 (33.9)	6 (60.0)	0.094
Urinary tract infection (n, %)	7 (4.2)	1 (10.0)	0.387
Wound infection (n, %)	29 (17.3)	4 (40.0)	0.072
Thrombembolism (n, %)	6 (3.6)	1 (10.0)	0.310
Intraabdominal abscess (n, %)	21 (12.5)	1 (10.0)	0.815
Pneumonia (n, %)	11 (6.5)	0 (0.0)	0.403
Reintubation (n, %)	14 (8.3)	0 (0.0)	0.342
Sepsis (n, %)	9 (5.4)	0 (0.0)	0.453
Acute kidney failure (n, %)	9 (5.4)	0 (0.0)	0.451
Insufficiency BDA (n, %)	1 (0.6)	0 (0.0)	0.807
Revision surgery (n, %)	24 (14.3)	3 (33.3)	0.122
Postoperative interventional therapy (n, %)	54 (32.1)	6 (60.0)	0.070
Postoperative conservative therapy (n, %)	128 (76.2)	9 (90.0)	0.314
Postoperative mortality (n, %)	4 (2.4)	0 (0.0)	0.622

p-values in bold print are statistically significant.

### Comparison of colonization of PDAC tissue versus benign tumors

Comparing PDAC with other tumor entities, we could find no difference in the total amount of a positive microbiological culture growth (26.7% vs. 29.5%, p = 0.669). Interestingly, we found significantly more cases of colonization with *E. faecium* in patients with PDAC compared to other tumor entities (8.9% vs. 1.1%, p = 0.018), but a trend towards less colonization with *E. faecalis* in PDAC patients (6.7% vs. 14.8%, p = 0.080). Concerning *E. coli*, we found no difference between PDAC and other tumor entities (p = 0.181). When comparing PDAC tumors of the pancreatic head with tumors of corpus or tail, we found no difference in the amount of microbiological colonization (27.9% vs. 22.7%, p = 0.631).

### Survival

**Figure 2:**
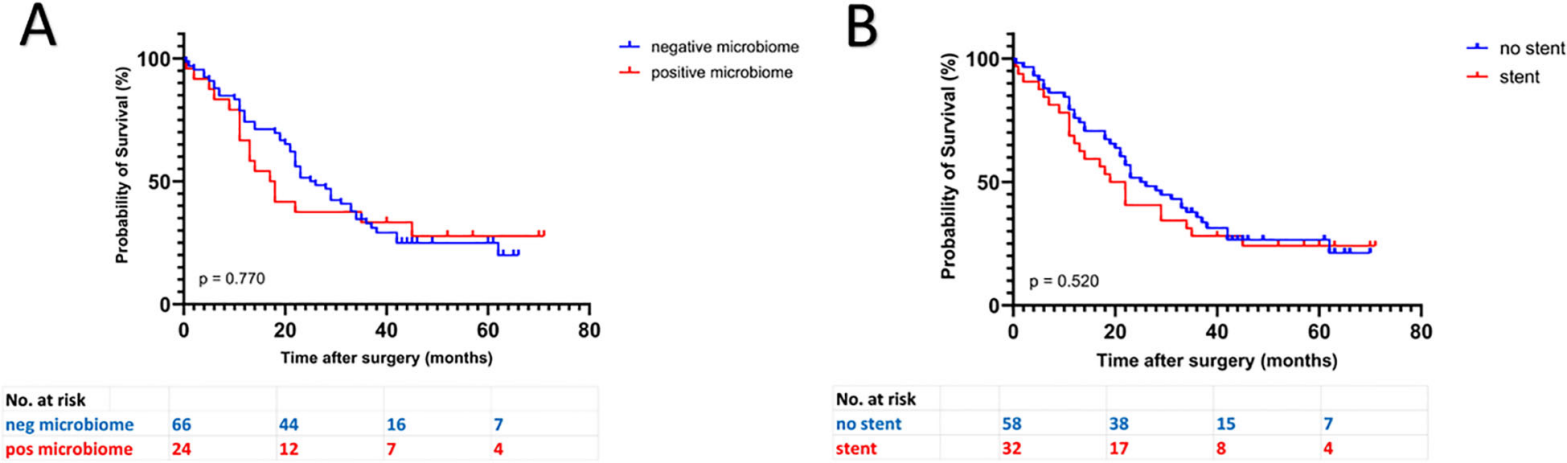
Kaplan Meier curves of PDAC patients. **(A)** negative vs. positive microbiome **(B)** stented vs. non-stented patients.

Concerning the 90 PDAC patients, we found a trend towards a worse survival in patients with microbiological colonization of the pancreatic tumor (17.5 months vs. 25.5 months), but without reaching statistical significance (p = 0.770). This can be observed in the Kaplan-Meier curves, which indicate a trend toward reduced survival in patients with positive microbiological findings, especially during the first 36 months following tumor resection. Subsequently, both survival curves run in parallel, suggesting that in long-term PDAC survivors, factors beyond microbial colonization may contribute to outcomes (**Figure 2A**). Comparing the survival curves of patients with and without preoperative bile duct stenting, we could find similar curves with stented patients tending to have a poorer survival (20.5 months vs. 25.5 months, p = 0.520) (**Figure 2B**).

### Comparison between conventional microbiological culture and 16S rDNA-sequencing

**Figure 3:**
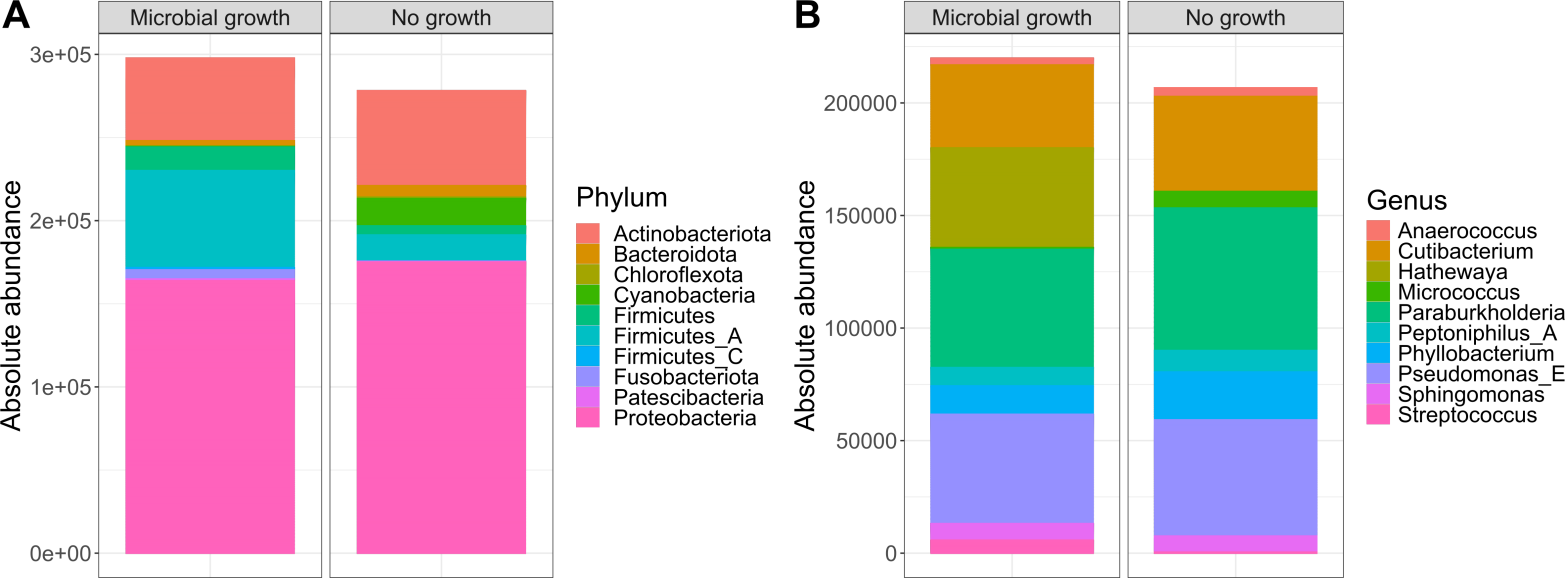
Abundant phyla and genera according to microbial growth. Ten most abundant phyla **(A)** and genera **(B)** from 16S rDNA-sequencing grouped by the presence of microbial growth in culture (n=10 per group). The y-axis represents absolute abundance.

**Figure 4:**
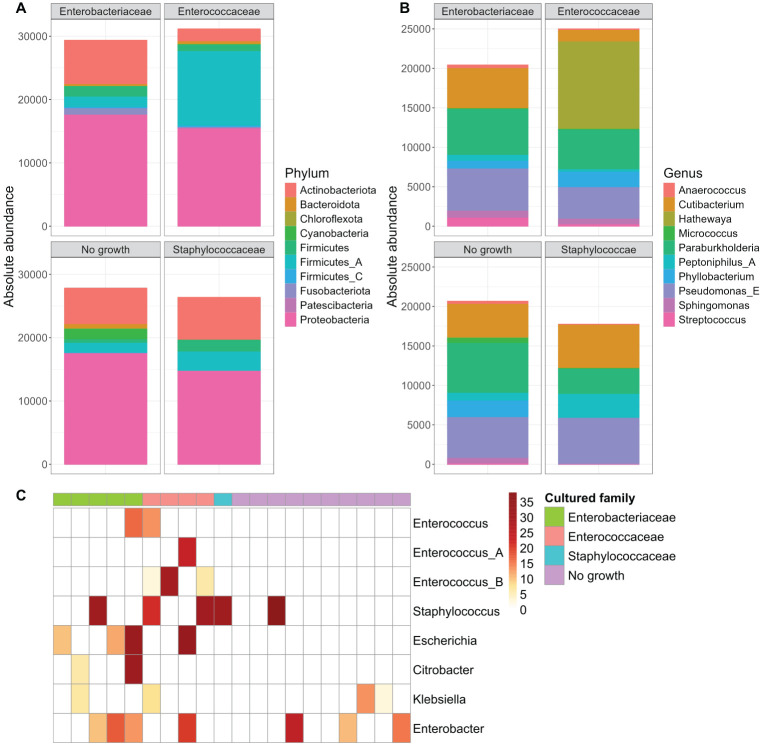
Abundant phyla and genera according to microbial growth in culture, grouped by family. Ten most abundant phyla **(A)** and genera **(B)** from 16S rDNA-sequencing grouped by the family of the cultured bacteria (Enterobacteriaceae n=5, Enterococcaceae n=4, Staphylococcaceae n=1) or by no observed growth (n=10). Read counts are normalized to the sample size (n) of each group. The y-axis represents absolute abundance. Heatmap of genera abundances that were cultured in the samples. Sample abundances were normalized using Cumulative Sum Scaling (CSS) **(C)**.

**Figure 5:**
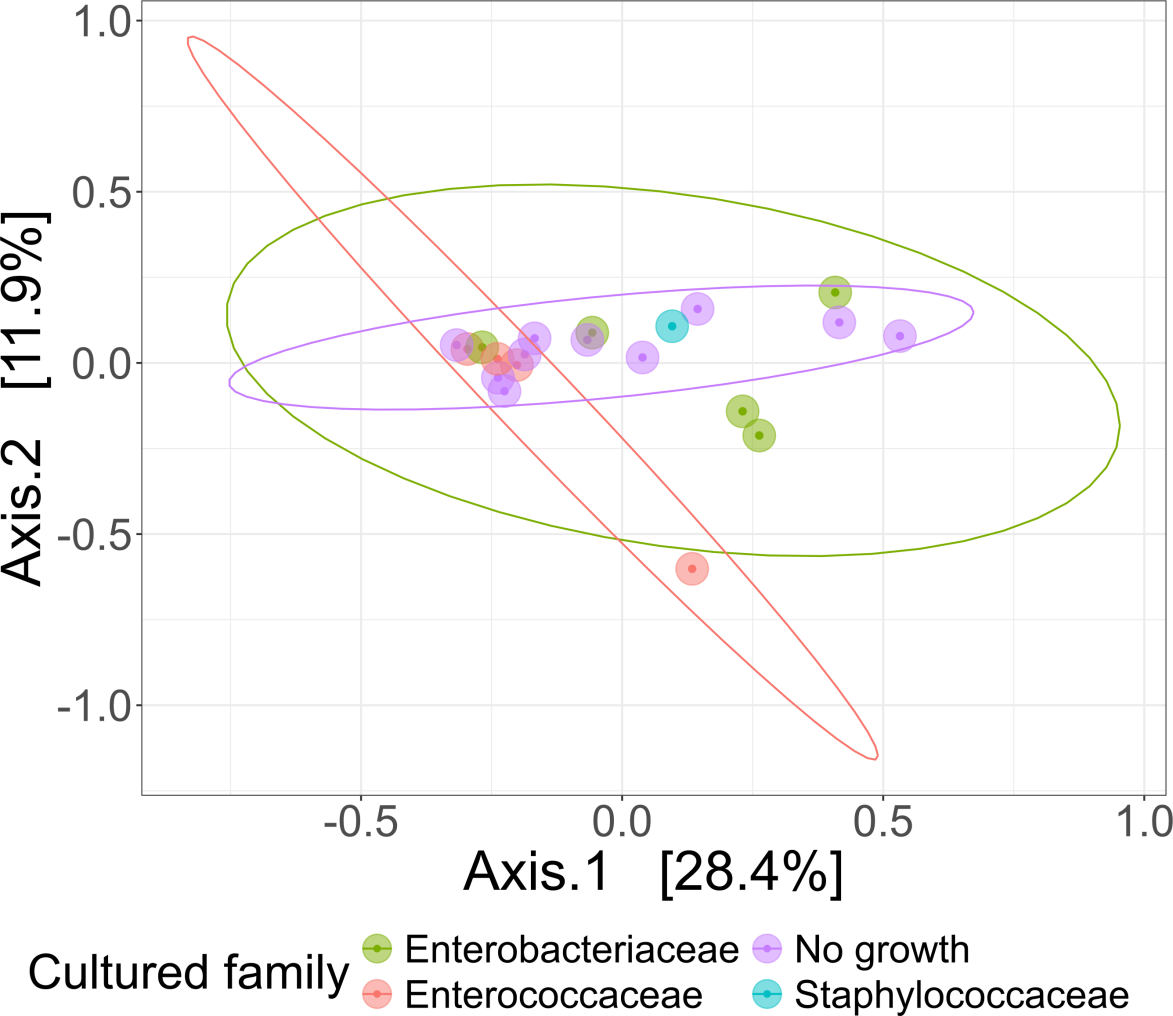
Comparison of beta diversities of microbial communities of patient samples, grouped based on their culture growth. PCoA visualization of bacterial community composition of different bacterial culture growth groups grouped by the family of the cultured bacteria (Enterobacteriaceae n=5, Enterococcaceae n=4, Staphylococcaceae n=1) or by no observed growth (n=10). Beta diversity measured with Bray-Curtis Dissimilarity Distances and visualized using Principal Coordinates Analysis (PCoA) plot.

**Figure 6:**
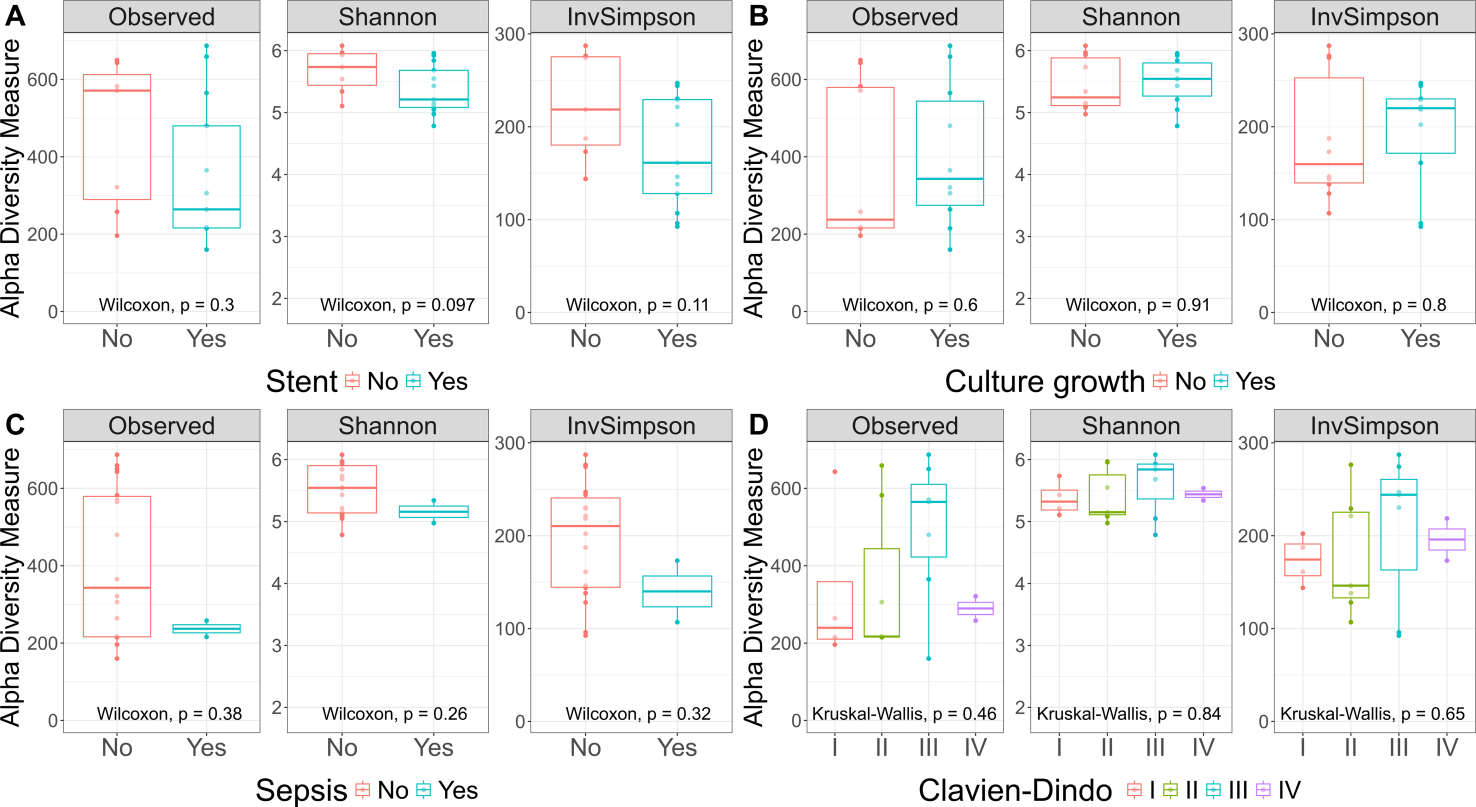
Alpha Diversity. Observed, Shannon, and InvSimpson Alpha Diversity. This boxplot showcases three alpha diversity indices across various patient groups: patients with and without stent **(A)**, patients with and without microbial growth in culture **(B)**, patients with post-surgery sepsis **(C)**, and Clavien-Dindo classification groups **(D)**. The central line within each box represents the median diversity value. The box spans the interquartile range (IQR), from the 25th to the 75th percentile, illustrating the middle 50% of the data. Whiskers extend to the smallest and largest values within 1.5 times the IQR from the quartiles. The Wilcoxon rank-sum test and the Kruskal-Wallis test showed no significant differences between the conditions for any of the alpha diversity indices.

**Figure 7:**
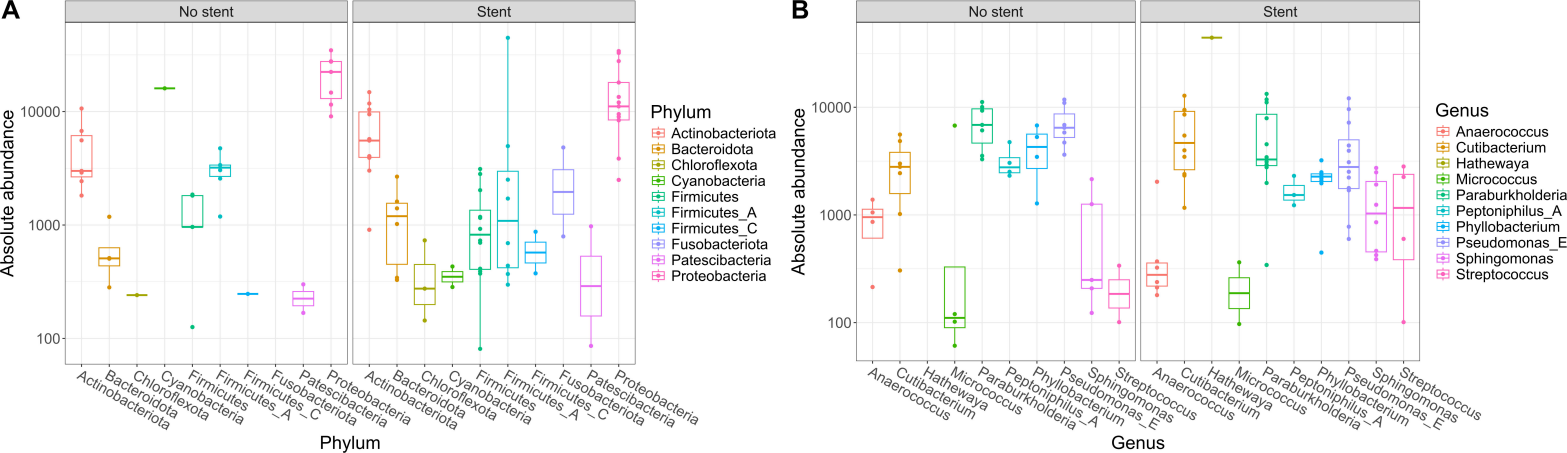
Abundant phyla and genera according to stent presence. Ten most abundant phyla **(A)** and genera **(B)** identified in 16S rDNA-sequencing grouped according to stent presence. The y-axis represents absolute abundance. The central line within each box represents the median abundance value. The box spans the interquartile range (IQR), covering the 25th to 75th percentiles, thus illustrating the middle 50% of the data. Whiskers extend to the smallest and largest values within 1.5 times the IQR from the quartiles. Each point on the plot corresponds to one sample.

As our study is one of the first studies analyzing the vital microbiome of pancreatic tumors via conventional microbiological culture of tumor tissue, we performed an analysis of 20 of our PDAC patient samples (10 with positive and 10 with negative microbial colonization in the conventional microbial culture) via 16S-rDNA sequencing in order to evaluate if the results in our cohort differ between conventional culture und 16S-rDNA sequencing. In this first comparison of conventional microbiological culture and 16S-rDNA sequencing in our cohort, there was a strong correlation between cultural growth of staphylococci and enterococci and the identification of these bacteria via sequencing. On the other hand, especially for *Enterobacteriaceae*, the most frequently found species in sequencing do not match the species growing in conventional culture. Moreover, even in tumor specimens without growth of bacteria in the conventional culture, we could detect several bacteria via sequencing. In contrast, with the exception of one tumor sample, all bacteria identified via cultural growth could be identified via sequencing too. The results concerning abundant phyla and genera according to microbial growth in culture are shown in **Figure 3** and grouped by the family of the cultured bacteria (*Enterobacteriaceae*, *Enterococcaceae*, *Staphylococcaceae*) or by no growth in **Figures 4A, B**. Most of the samples (4 out of 5) in which *Enterococcus* spp. were identified through 16S rDNA sequencing also exhibited growth of *Enterococcus* in culture. Conversely, multiple taxa from *Enterobacteriaceae* and *Staphylococcaceae* identified by 16S-rDNA sequencing were not detected in culture (**Figure 4C**). The broader microbial community structure was not substantially influenced by the cultured bacteria, as shown by the minimal impact on beta diversity (**Figure 5**). We further investigated the bacterial community structure in pancreatic tumor samples by examining the alpha diversity, which captures both the richness (variety of bacterial taxa) and evenness (the distribution of their abundances). Alpha diversity was assessed using the observed species, Shannon index and inverse Simpson (InvSimpson) index. Here, we found no difference between patients with positive or negative microbiological growth in the conventional culture (Observed *P* = 0.6, Shannon *P* = 0.91, InvSimpson *P* = 0.8). However, in patients with preoperatively inserted bile duct stents, there seems to be a trend towards a reduced alpha diversity in comparison to patients without bile duct stents, but without reaching statistical significance (Observed *P* = 0.3, Shannon *P* = 0.097, InvSimpson *P* = 0.11). We found no differences in alpha-diversity between patients with and without postoperative sepsis (Observed *P* = 0.38, Shannon *P* = 0.26, InvSimpson *P* = 0.32) or between different Clavien-Dindo stages (Observed *P* = 0.46, Shannon *P* = 0.84, InvSimpson *P* = 0.65). Results concerning alpha diversity are shown in **Figure 6**. Moreover, via 16S-rDNA sequencing, we could find a shift towards an increase in *Cutibacterium* in patients with bile duct stents. *Cutibacterium* represent typically stent-associated bacteria that grow hardly in conventional culture, so that we couldn´t find them via conventional culture, but verify them via sequencing especially in the stented patients. An overview of the abundant phyla and genera according to stent presence is given in **Figure 7**.

## Discussion

Pancreatic cancer is one of the most common causes of cancer mortality in developed countries (Raimondi et al., 2009). During recent years, the pancreas´ microbiome turned in the focus of cancer research (Picardo et al., 2019), as alterations in the microbiome may lead to disease development and progression (Frost et al., 2022). In cancers not directly linked to known oncogenic microbes (e.g. Helicobacter pylori, HPV, EBV or HBV), accumulating evidence suggests that microbial - particularly bacterial -colonization of tumor tissue actively contributes to the tumor microenvironment (Sepich-Poore et al., 2021). Recent studies employing advanced sequencing technologies, such as those by Galeano Niño et al., demonstrate that the intratumoral microbiota is organized into distinct microniches and functionally impacts tumor biology by activating oncogenic pathways (e.g. JUN/FOS) and immune-suppressive mechanisms (e.g. JAK–STAT), thereby promoting cancer progression (Galeano Niño et al., 2022). This microbial advantage in tumor progression may stem from enhanced survival benefits under fluid shear stress in the circulatory system, as observed in bacterial-colonized tumor cells (Bullman et al., 2017; Fu et al., 2022). In studies examining intratumoral bacteria in pancreatic cancer, Geller et al. demonstrated bacterial colonization in 76% of human PDAC samples, predominantly by Gammaproteobacteria. These bacteria were shown to inactivate the chemotherapy drug gemcitabine through cytidine deaminase (CDDL) activity, thereby promoting treatment resistance, an effect that was reversible with antibiotic administration (Geller et al., 2017). Furthermore, the correlation between metabolic and genetic subtypes in pancreatic cancer highlights the need to further investigate microbiota-metabolism interactions. Notably, early-stage tumors exhibit elevated serum polyamines, a microbial-linked metabolite that could serve as a noninvasive diagnostic biomarker for pancreatic cancer (Mendez et al., 2020; Zhang et al., 2020; Ling and Kalthoff, 2021).

The role of gut microbiota in modulating the efficacy of anticancer treatment and promoting resistance to chemotherapeutic drugs or immune checkpoint inhibitors has been known for several years (Cheng et al., 2020). Recently, it could be shown that pancreatic cancer tissue comprises a more abundant microbiome compared to normal pancreatic tissue both in humans as well as in mice and that selected bacteria are differentially increased in pancreatic cancer tissue, compared to the gut microbiome (Pushalkar et al., 2018). In the first prospective evaluation of our patient cohort over a three-year period, we could prove a microbiological colonization of pancreatic tissue in almost a third of our patients. This microbiological colonization in our collective seems to be promoted by preoperatively inserted bile duct stents as we found significantly more microbiological colonization of the pancreatic tumor in stented patients. The influence of preoperative bile duct stenting on the biliary microbiome was already shown earlier (Scheufele et al., 2017). Alterations of the microbiome in patients undergoing preoperative stent placement were also described by Langheinrich et al (Langheinrich et al., 2020). In this cohort, an increased rate of POPF in stented patients was observed (Langheinrich et al., 2020). We, however, found no difference in fistula rates in our patient cohort although the rate of stented patients was significantly higher in the group with a positive microbiological colonization. Nalluri et al. found a significantly higher rate of positive bacterial colonization of pancreatic tumor tissue in patients with preoperative bile duct stenting, too (Nalluri et al., 2021). Moreover, they observed an association of neoadjuvant chemotherapy with specific alterations of the intra-tumor bacteria in PDAC patients (Nalluri et al., 2021). The alteration of the biliary microbiome by neoadjuvant chemotherapy in PDAC patients was also described by Goel et al., showing significantly more enterococci and Klebsiella in the bile of these patients, but without influence on surgical site infections or POPF (Goel et al., 2019). Similar results, namely an influence of neoadjuvant chemotherapy on the biliary microbiome, but without impact on infectious postoperative outcomes, were found by Nadeem et al (Nadeem et al., 2021). Actually, in our patient collective, we observed an association of neoadjuvant chemotherapy and positive microbiological findings in our evaluation of the first 60 patients after a one-year period. However, in analyzing the entire patient collective after this three-year period, we couldn´t find a significant association between neoadjuvant chemotherapy and microbiological colonization of the pancreatic tumor any more. Another study could show that the bacteria that coexist in the tumor tissues of pancreatic and biliary tract cancer were relatively common to those localized in pancreatic and gastric juice, suggesting that they might originate from these environments (Okuda et al., 2022). Bacterial spread to the pancreas by blood stream, transmurally from the colon or by reflux into the pancreatic duct could already be shown in an animal model in the early nineties of the last century (Widdison et al., 1994). A Chinese review from 2019 showed a summary of microbes influencing tumor development and progression in pancreatic cancer, mentioning amongst others enterococcus species and *E. coli* as important bacteria leading to the development of PDAC (Wei et al., 2019); these species were also frequent in our patient collective. Especially *E. coli* seems to be associated with more complications following pancreatic surgery, as we could find a significantly higher rate of postpancreatectomy hemorrhage in these patients. In addition, we observed a trend towards more CR-POPF and towards a higher rate of DGE in the presence of *E. coli* in the pancreatic tumor. Riquelme et al. could show a different microbiome in resectable PDAC patients with short- and long-term survival by 16S-rRNA sequencing, so that the microbiome seems to influence the hosts immune response against tumor cells and thereby the long-term outcome of PDAC patients (Riquelme et al., 2019). Especially *Pseudoxanthomonas, Saccharopolyspora* and *Streptomyces* spp. were associated with long-term survival in this cohort (Riquelme et al., 2019). In our patient cohort, we could find a trends towards a negative influence of a microbiological colonization of the pancreas tumor in PDAC patients on long-term survival in these patients, but without reaching statistical significance. This may be attributed to the relatively small number of PDAC patients with positive microbiological findings in our cohort; therefore, further studies with a larger patient population are needed. Moreover, the RNA-sequencing method used by Riquelme et al. might be more precise in revealing microbiological findings than the standard microbiological culture of tumor tissue used in our study. In our small collective of 20 PDAC patients with additional 16S-rDNA sequencing, we could find a trend towards a reduced alpha diversity in patients with preoperatively inserted bile duct stent. As we could additionally show a trend towards a reduced survival in stented patients, this might support the findings of Riquelme et al. in terms of a higher alpha-diversity in long-term survivors (Riquelme et al., 2019). Furthermore, in our patient cohort, we could find significantly more patients with advanced lymph node involvement (N2-stages) in the group with a positive microbiological culture of the pancreatic tumor, suggesting a more aggressive tumor type in these patients, consequently leading to a poorer survival in this group. A Korean group used extracellular vehicles and 16S-rRNA sequencing to identify the composition and diversity of the microbiome in tissues of pancreatic cancer (Jeong et al., 2020). This group observed differences in the microbiome depending on the rate of lymph node metastasis as well. Moreover, a change in the microbiome depending on the primary tumor size was described (Jeong et al., 2020). In our collective, however, we didn´t observe any correlation between tumor size and the rate of microbiological colonization, at least not by using conventional microbiological culture techniques. Here, further studies with 16S-rRNA sequencing of a larger collective might be warranted.

There are several limitations of our study. First, it is a single center study, even though of a University Hospital. Second, we predominantly performed the standard microbiological cultivation of pancreatic tumor tissue which may have led to less sensitive results concerning bacterial and fungal colonization, compared to 16S-rDNA sequencing methods. However, this study provides an insight of microbiological species associated with pancreatic tumor tissue and their potential influence on patient outcome. Further studies are needed to reveal a closer look on the PDAC microbiome and its influence on oncological long-term outcome.

## Data Availability

The raw sequences generated in this project were deposited in the European Nucleotide Archives (ENA, https://www.ebi.ac.uk/ena) and are available under study number PRJEB89295 and samples accession numbers ERS24440838-ERS24440857.
