# Supplementary figures and images for: Microbiological colonization of the pancreatic tumor affects postoperative complications and outcome after pancreatic surgery
Source: Front Cell Infect Microbiol. 2025 May 30;15:1521952. doi: 10.3389/fcimb.2025.1521952 (PMC12162974; doi:10.3389/fcimb.2025.1521952)

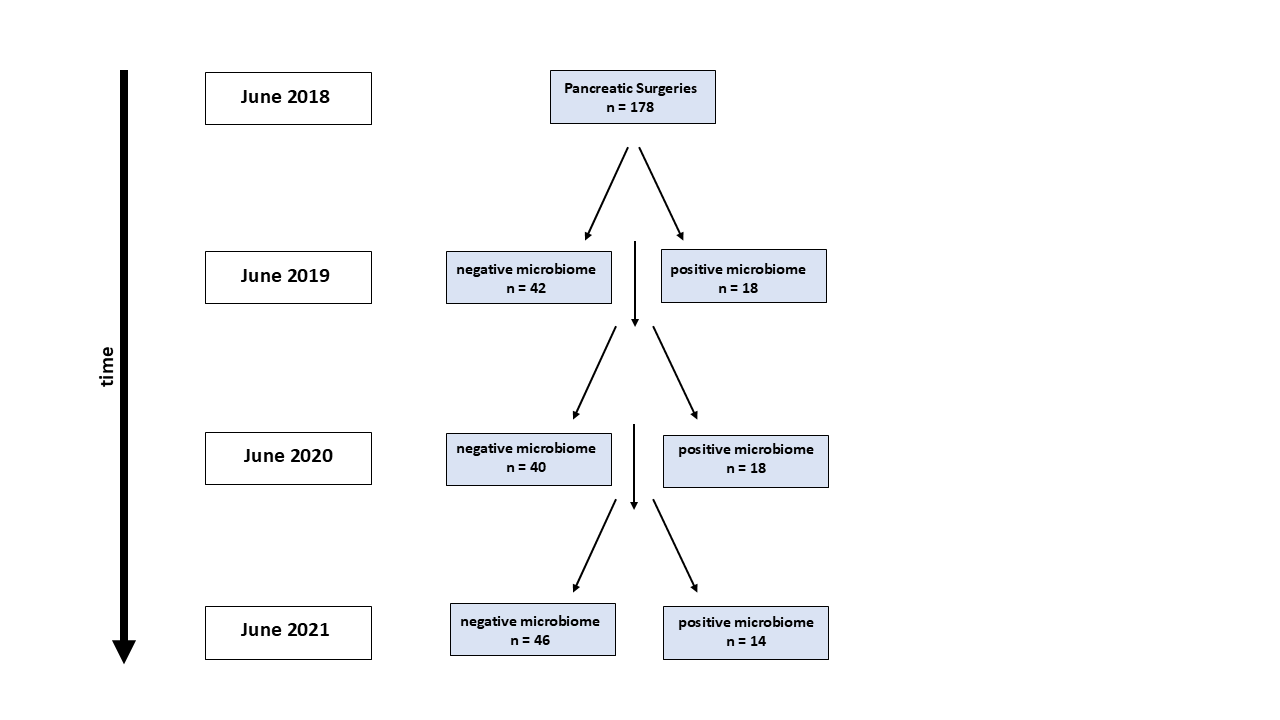

Supplement: Supplementary file 1 [file Image1.tif]
