# Supplementary material for: Microbiological colonization of the pancreatic tumor affects postoperative complications and outcome after pancreatic surgery
Source: Front Cell Infect Microbiol. 2025 May 30;15:1521952. doi: 10.3389/fcimb.2025.1521952 (PMC12162974; doi:10.3389/fcimb.2025.1521952)
Supplement: Supplementary file 2 [file DataSheet1.pdf]

Supplementary Tables

Microbiological colonization of the pancreatic tumor affects postoperative complications and outcome after pancreatic surgery

Esther Anna Biesel[1], Johanna Sundheimer[1], Mohamed Tarek Badr[2], Sara Posadas-Cantera[2], Sophia Chikhladze[1], Stefan Fichtner-Feigl[1], Uwe Alexander Wittel[1]

<u>**Supplementary Table 1:**</u> *Baseline characteristics and intraoperative parameters in association with colonization with E. coli at the time of surgery*

	No **evidence of** *E. coli* **on pancreatic tumor (n = 168)**	**Colonization with** *E. coli* **(n = 10)**	**p-value**
**Age**, years (median, range)	66 (20 – 86)	72 (23 – 84)	0.764
**Sex** (n, %)			0.198
- male	100 (59.5)	8 (80.0)	
- female	68 (40.5)	2 (20.0)	
**BMI,** kg/m2 (median, range)	25.2 (16.2 – 43.9)	27.5 (21.8 – 64.3)	**< 0.001**
**ASA stadium** (n, %)			
- ASA 1	1 (0.6)	0 (0.0)	0.807
- ASA 2	46 (27.4)	4 (40.0)	0.388
- ASA 3	114 (67.9)	6 (60.0)	0.607
- ASA 4	7 (4.2)	0 (0.0)	0.510
**Comorbidities** (n, %)	155 (92.3)	6 (60.0)	**< 0.001**
- Coronary heart disease	16 (9.5)	2 (20.0)	0.286
- Hypertension	97 (57.7)	5 (50.0)	0.631
- Pulmonary disease	36 (21.4)	0 (0.0)	0.101
- Renal disease	18 (10.7)	0 (0.0)	0.275
- Liver disease	27 (16.1)	0 (0.0)	0.169
- Diabetes mellitus	49 (29.2)	3 (30.0)	0.955
Alcohol abuse (n, %)	32 (19.2)	2 (20.0)	0.948
Nicotin abuse (n, %)	62 (36.9)	3 (30.0)	0.812
**Neoadjuvant therapy** (n, %)	18 (10.7)	1 (10.0)	0.943
**Bile duct stent preoperative** (n, %)	49 (29.2)	8 (80.0)	**< 0.001**

Preoperative leucocytes*10³/µl (median, range)	7.1 (2.9 – 17.4)	7.1 (4.6 – 11.8)	0.987
Preoperative hemoglobin, g/dl (median, range)	13.0 (8.9 – 20.4)	13.4 (8.4 – 14.9)	0.843
Preoperative thrombocytes*10³/µl (median, range)	257.0 (41 – 589)	275.5 (195 – 344)	0.846
Preoperative creatinine, mg/dl (median, range)	0.82 (0.4 – 2.2)	0.85 (0.4 – 1.3)	0.750
Preoperative bilirubine, mg/dl (median, range)	0.6 (0.2 – 33.8)	0.85 (0.2 – 1.9)	0.279
Preoperative serum amylase, U/l (median, range)	25.0 (2.0 – 674.0)	31.0 (9.0 – 211.0)	0.267
Preoperative INR (median, range)	1.02 (0.90 – 1.85)	0.99 (0.95 – 1.10)	0.302
**Indication for surgery** (n, %)			
- PDAC	87 (51.8)	3 (30.0)	0.181
- periampullary carcinoma	22 (13.1)	5 (50.0)	**0.002**
- IPMN	13 (7.7)	0 (0.0)	0.361
- Chronic pancreatitis	14 (8.3)	2 (20.0)	0.210
- Neuroendocrine tumor	15 (8.9)	0 (0.0)	0.323
- Other malign	2 (1.2)	0 (0.0)	0.729
- Other benign	15 (8.9)	0 (0.0)	0.323
**Duration of surgery**, minutes (median, range)	372.5 (78 – 722)	369.5 (162 – 446)	0.665
**Surgical technique** (n, %)			
- pancreatoduodenectomy (open)	56 (33.3)	4 (40.0)	0.665
- pancreatoduodenectomy (min. invasive)	74 (44.0)	6 (60.0)	0.325
- distal pancreatectomy (open)	5 (3.0)	0 (0.0)	0.580
- distal pancreatectomy (min. invasive)	17 (10.1)	0 (0.0)	0.290
- total pancreatectomy	7 (4.2)	0 (0.0)	0.510
- laparoscopic enucleation	1 (0.6)	0 (0.0)	0.807
- other surgery	8 (4.8)	0 (0.0)	0.480
**Transfusion of red blood cells intraoperatively**, ml (mean, SD)	66.5 (300.1)	0 (0.0)	0.487
**Resection margin negative (R0)** (n, %)	106 (84.4)	7 (87.5)	0.836
**Histopathological classification** (n, %) (n = 130)			
- T1	25 (20.5)	2 (25.0)	0.761
- T2	47 (38.5)	4 (50.0)	0.520
- T3	43 (35.2)	2 (25.0)	0.555
- T4	7 (5.7)	0 (0.0)	0.486
- N0	52 (43.0)	3 (37.5)	0.762
- N1	38 (31.4)	1 (12.5)	0.259
- N2	31 (25.6)	4 (50.0)	0.133

Supplementary Table 2: *Complications depending on preoperative biliary stenting*

	**Patients *without* preoperative biliary stenting (n = 121)**	**Patients *with* preoperative biliary stenting (n = 57)**	**p-value**
Delayed Gastric Emptying (DGE) (n, %)	45 (37.5)	22 (40.0)	0.752
Postpancreatectomy Hemorrhage (PPH B/C) (n, %)	11 (9.1)	7 (12.3)	0.510
Pancreatic fistula (CR-POPF) (n, %)	41 (33.9)	22 (38.6)	0.540
Urinary tract infection (n, %)	5 (4.1)	3 (5.3)	0.734
Wound infection (n, %)	17 (14.0)	16 (28.1)	**0.025**
Thrombembolism (n, %)	5 (4.1)	2 (3.5)	0.842
Intraabdominal abscess (n, %)	16 (13.2)	6 (10.5)	0.610
Pneumonia (n, %)	9 (7.4)	2 (3.5)	0.310
Reintubation (n, %)	11 (9.1)	3 (5.3)	0.376
Sepsis (n, %)	6 (5.0)	3 (5.3)	0.931
Acute kidney failure (n, %)	4 (3.3)	5 (8.9)	0.113
Insufficiency BDA (n, %)	0 (0.0)	1 (1.8)	0.144
Revision surgery (n, %)	16 (13.2)	11 (19.6)	0.269
Postoperative interventional therapy (n, %)	37 (30.6)	23 (40.4)	0.198
Postoperative conservative therapy (n, %)	85 (70.2)	52 (91.2)	**0.002**
Postoperative mortality (n, %)	2 (1.7)	2 (3.5)	0.436
Length of hospital stay, days (median,range)	14 (5 – 55)	16 (9 – 73)	0.917
Length of ICU stay, days (median, range)	5 (2 – 38)	5 (3 – 41)	0.657
